# Mortality Risks after Two Years in Frail and Pre-Frail Older Adults Admitted to Hospital

**DOI:** 10.3390/jcm12093103

**Published:** 2023-04-24

**Authors:** Guillermo Cano-Escalera, Manuel Graña, Jon Irazusta, Idoia Labayen, Ana Gonzalez-Pinto, Ariadna Besga

**Affiliations:** 1 Department of Computer Science and Artificial Intelligence, University of the Basque Country (UPV/EHU), 20018 Donostia-San Sebastian, Spain; 2 Computational Intelligence Group, University of the Basque Country (UPV/EHU), 20018 Donostia-San Sebastian, Spain; 3 Department of Physiology, Faculty of Medicine and Nursing, University of the Basque Country (UPV/EHU), 48940 Bilbao, Spain; 4 BioCruces Health Research Institute, 48903 Barakaldo, Spain; 5 Institute for Innovation & Sustainable Development in Food Chain (IS-FOOD), Public University of Navarra, 31006 Pamplona, Spain; 6 BioAraba, Health Research Institute, Department of Medicine, Hospital Universitario de Araba, 01004 Vitoria, Spain; 7 Biomedical Research Centre in Mental Health Network (CIBERSAM), 28029 Madrid, Spain

**Keywords:** frailty, frail, pre-frail, fried frailty scale, mortality, survival

## Abstract

Background: Frailty is characterized by a progressive decline in the physiological functions of multiple body systems that lead to a more vulnerable condition, which is prone to the development of various adverse events, such as falls, hospitalization, and mortality. This study aims to determine whether frailty increases mortality compared to pre-frailty and to identify variables associated with a higher risk of mortality. Materials: Two cohorts, frail and pre-frail subjects, are evaluated according to the Fried phenotype. A complete examination of frailty, cognitive status, comorbidities and pharmacology was carried out at hospital admission and was extracted through electronic health record (EHR). Mortality was evaluated from the EHR. Methods: Kaplan–Meier estimates of survival probability functions were calculated at two years censoring time for frail and pre-frail cohorts. The log-rank test assessed significant differences between survival probability functions. Significant variables for frailty (*p* < 0–05) were extracted by independent sample *t*-test. Further selection was based on variable significance found in multivariate logistic regression discrimination between frail and pre-frail subjects. Cox regression over univariate *t*-test-selected variables was calculated to identify variables associated with higher proportional hazard risks (HR) at two years. Results: Frailty is associated with greater mortality at two years censoring time than pre-frailty (log-rank test, *p* < 0.0001). Variables with significant (*p* < 0.05) association with mortality identified in both cohorts (HR 95% (CI in the frail cohort) are male sex (0.44 (0.29–0.66)), age (1.05 (1.01–1.09)), weight (0.98 (0.96–1.00)), and use of proton-pump inhibitors (PPIs) (0.60 (0.41–0.87)). Specific high-risk factors in the frail cohort are readmission at 30 days (0.50 (0.33–0.74)), SPPB sit and stand (0.62 (0.45–0.85)), heart failure (0.67 (0.46–0.98)), use of antiplatelets (1.80 (1.19–2.71)), and quetiapine (0.31 (0.12–0.81)). Specific high-risk factors in the pre-frail cohort are Barthel’s score (120 (7.7–1700)), Pfeiffer test (8.4; (2.3–31)), Mini Nutritional Assessment (MNA) (1200 (18–88,000)), constipation (0.025 (0.0027–0.24)), falls (18,000 (150–2,200,000)), deep venous thrombosis (8400 (19–3,700,000)), cerebrovascular disease (0.01 (0.00064–0.16)), diabetes (360 (3.4–39,000)), thyroid disease (0.00099 (0.000012–0.085)), and the use of PPIs (0.062 (0.0072–0.54)), Zolpidem (0.000014 (0.0000000021–0.092)), antidiabetics (0.00015 (0.00000042–0.051)), diuretics (0.0003 (0.000004–0.022)), and opiates (0.000069 (0.00000035–0.013)). Conclusions: Frailty is associated with higher mortality at two years than pre-frailty. Frailty is recognized as a systemic syndrome with many links to older-age comorbidities, which are also found in our study. Polypharmacy is strongly associated with frailty, and several commonly prescribed drugs are strongly associated with increased mortality. It must be considered that frail patients need coordinated attention where the diverse specialist taking care of them jointly examines the interactions between the diversity of treatments prescribed.

## 1. Introduction

Frailty is a state mostly prevalent in older adults that emerges when many physiological systems have a gradual functional decline converging to vulnerability conditions [1,2], which may reach a point of no return that leads to death [3]. Frail persons are at greater risk of adverse health outcomes, including falls, hospitalization, and mortality [4,5]. Although frailty is not synonymous with comorbidity or disability, there is growing evidence that it can be considered as a phenotype [6]. Frailty may occur at all adult ages [7], but it is closely related to aging, hence frailty prevalence is more likely to increase as the population ages [8].

Frailty is being recognized as an emerging global health burden, and the prevalence of frailty is expected to increase along with the rapid growth of the old-age segment of the population [9]. Medical costs increase in association with frailty prevalence [10], which ranges from 4% to 17% [11] in community living adults aged 65 years or more. Prevalene of pre-frailty ranges from 19% and 53% in several studies.

These differences between the prevalence estimates depend on the definition of frailty, which may be based on physical markers or on the use of a broader multidimensional approach, and the specific population studied [12].

In recent years, multiple instruments have been developed to define frailty and its dimensions [13,14], aiming to make it objectively classifiable [15]. Fried et al. [16] initially hypothesized some clinical presentations of frailty, which were later operationalized in a diagnostic instrument, known as the frailty phenotype, which was validated in a cardiovascular health study [6]. Subsequently, Rockwood et al. [17] conducted a study to develop and validate the frailty index, to produce useful predictive information. These two instruments, despite being different in terms of their analysis of frailty, should be considered complementary for the study and diagnosis of frailty [18].

The clinical presentation of frailty is very heterogeneous; thus, well-structured strategies for the provision of frailty care are needed to reduce its personal, social, and economic burden. Systematic case identification may be key for good clinical practice, as well as for appropriate prevention and treatment of frailty [19].

During hospital stay [20], patient recovery is greatly determined by detection of clinically induced frailty and subsequent treatments. Interventions to manage the adverse consequences of frailty try either to address the dependence and disability risks or to treat symptoms of underlying conditions [21]. On the other hand, good management of frailty conveys the development of coping strategies, necessary to control possible stressors or to reduce the scope of their impact after hospital discharge [22].

Early in the COVID-19 pandemic, a strong association of frailty scale and mortality during hospital admittance was reported for older patients [23,24]. Frailty was associated with high mortality in patients aged 75+ years admitted at the hospital and diagnosed with COVID-19 in England [25]. As much as 50% of frail patients that had positive identification of the virus died before 28 days, while 25–35% of those that had COVID-19 diagnosis but not positive identification of the virus died in the same period. In Italy, frailty was associated with increasing COVID-19 mortality in long-term care [26] and in hospital admittance [27]. Paradoxically, a systematic review [28] did not show a relation between COVID-19 mortality at the hospital and frailty, because non frail patients were prioritized to receive invasive mechanical ventilation that resulted in much worse outcomes. A cohort study [29] confirms these findings. However, a living review [30] found increasing association between frailty and COVID-19 mortality, as did another systematic review [31] in almost all studies revised.

Our study contributes to the understanding of the complex relationship between frailty and risk of mortality in the context of hospital admissions of older adults [32] in a pre-pandemic period. We have collected a large number of variables per patient at hospital admission that are assessed for their association with increased mortality risk.

## 2. Materials and Methods

### 2.1. Study Design and Subjects

**Figure 1:**
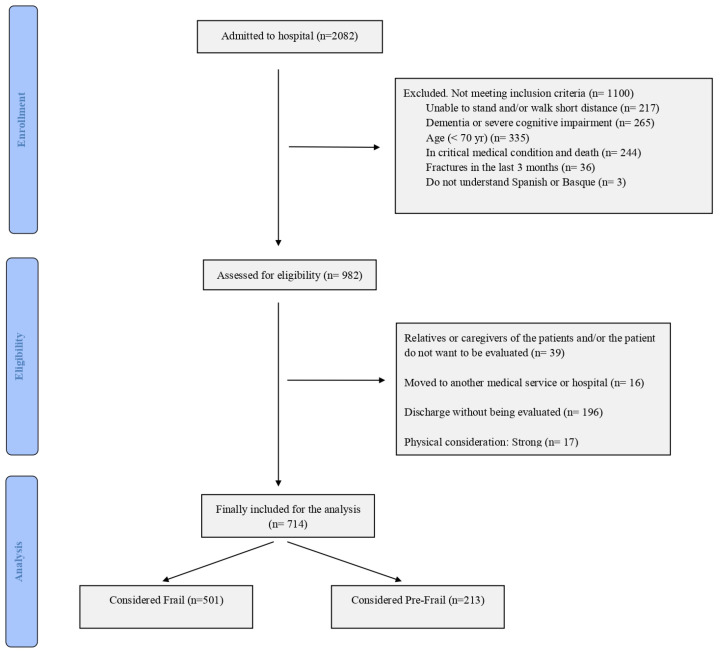
Flow diagram of the recruitment process.

The anonymized and cleaned dataset has been published in Zenodo [33]. Figure 1 provides a flow chart of the patient recruitment process. Patients entering the University Hospital of Alava (UHA) through internal medicine and neurology services were evaluated for frailty using Fried’s frailty index (FFI) [6]. Stabilized and able patients were considered for recruitment during the time interval from September 2017 up to September 2018. Institutional EHR was queried to detect deceased (non-survivors) patients until January 2021. Patients above 70 years with a score above 20 in the Spanish version of MMSE were able to walk without aids as well as elemental physical tests, following simple instructions were presented with the informed consent for recruitment.

The cutoff value on the MMSE is lower than specified when detecting cognitive dysfunction in highly educated persons [34] that use their education resources to hide their cognitive issues. In addition, the MMSE cutoff value to indicate dementia may be dependent on ethnic and cultural factors [35]; thus, it must be used with caution to signal dementia [36]. An experienced clinical researcher (AB) with knowledge on the specific responses of the local population helped to set the cutoff value that identified cognitively healthy older individuals whose cognitive abilities were at the mean level or slightly lower than expected based on age.

Criteria for exclusion are detailed in the document published to avoid self-plagiarism in the editorial [33]. The cohort is composed of 501 frail, 213 pre-frail, and 17 robust patients, which were excluded from this study. Functional status evaluation included the Short Physical Performance Battery (SPPB) [37], FFI, Barthel’s index score (BIS) [38], and the number of falls during the month previous to hospital admission. The Mini-Nutritional Assessment—Short Form (MNA-SF) is used as an indicator of malnutrition risks [39,40]. Mental status was assessed by the Pfeiffer’s Brief Screening Test for Dementia (PBSTD) [41,42]. Sociodemographic, clinical (comorbidities), and pharmacological data were queried from the EHR. Imputation of missing values followed consultation with clinicians and revisiting the EHR. A threshold for discarding a variable was set at 20% of cases.

### 2.2. Statistical Methods

Variable selection prior to survival analysis was carried out on the Jasp package (https://jasp-stats.org accessed on 1 January 2023). Selection of statistically significant variables (p<0.05) for frailty was carried out first by univariate Independent Samples *t*-test. Over the selected variables, a further multivariate significant variable (p<0.05) selection was carried out applying logistic regression discriminating frail from pre-frail subjects. For survival analysis, we used Rstudio 1.2 and R 3.6.3 (www.r-project.org, accessed on 1 March 2022) with packages HSAUR2, Survival, and Survminer. Kaplan–Meier estimates, at 2 years, censoring time of survival probability functions were computed [43,44,45]. The statistical significance (p<0.05) of differences between frail versus pre-frail survival probability functions was assessed by the Log-rank test [45]. Cox’s regression [45,46] provided separate hazard risks (HR) at 2 years, censoring time multivariate estimates for the frail and the pre-frail cohorts. Schoenfeld residuals, significant at p<0.05, assess the assumption of proportional hazards for all Cox regression analyses.

## 3. Results

**Figure 2:**
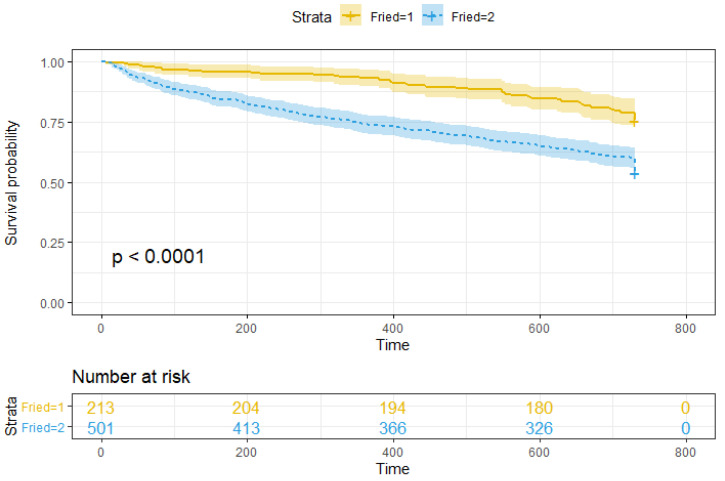
Kaplan–Meier survival curves for frail and pre-frail patients. Data are censored after 2 years.

Demographics and descriptive statistics of the sample can not be reproduced here due to self-plagiarism by the first author’s PhD Thesis as mandated by the editorial, so the reader is referred to the short document published along with the data [33]. Figure 2 compare the survival probability Kaplan–Meier estimates of the frail cohort versus pre-frail cohort in 2 years. Log-rank test *p*-value is strongly significant $p=3\times 10^{8}$, hence frail patients had a worse prognosis for survival at 2 years compared to the pre-frail subjects.

Due to the large number of variables, multivariate logistic regression over all of them was unfeasible. Therefore, we first carried out variable selection based on independent samples univariate *t*-test. Supplementary Table S1 reports the results of these tests. We selected significant variables (p<0.01) to carry out a multivariate logistic regression discriminating frail versus pre-frail subjects whose results are reported in full in Table S2 of the Supplementary Materials. Table 1 contains the statistics of significant variables found by logistic regression.

Supplementary Figures S1 and S2 graphically display the HR results of the multivariate Cox’s regression at 2-year censoring computed over all variables selected by independent samples *t*-test.

Table 2 and Table 3 summarize the variables with significant HR for the frail and pre-frail cohorts, respectively, at 2 years censoring times.

Variables associated with significant HR identified in both cohorts were age [frail (1.05 (1.01–1.09)); pre-frail (1.7 (1.3–2.2))], male sex [frail (0.44 (0.29–0.66)); pre-frail (0.005 (0.00011–0.23))], weight [frail (0.98 (0.96–1.00)); pre-frail (1.3 (1.1–1.5))], congestive heart failure [frail (0.67 (0.46–0.98)); pre-frail (42 (1.3–1300))], and the use of PPIs [frail (0.60 (0.41–0.87)); pre-frail (0.062 (0.0072–0.54))].

Specific variables associated with significant HR in the frail cohort were readmission before 30 days after discharge (0.50 (0.33–0.74)), SPPB time to sit and stand up (0.62 (0.45–0.85)), and the use of antiplatelets (1.80 (1.19–2.71)), quetiapine (0.31 (0.12–0.81)), and paracetamol 1.45 (1.00–2.08).

Specific variables associated with significant HRs in the pre-frail cohort were live in your own house (120 (2.2–7100)), Barthel’s score (120 (7.7–1700)), Pfeiffer score (8.4 (2.3–31)), MNA (1200 (18–88,000)), MNA weight loss 3 months (590 (11–32,000)), MNA mobility (590 (11–32,000)), MNA acute disease 3 months (4.7 × $10^{4}$ (8.7 × $10^{06}$–0.026)), vision loss (0.042 (0.0037–0.49)), constipation (0.025 (0.0027–0.24)), history of falls (18,000 (150–2,200,000)), deep venous thrombosis (8400 (19–3,700,000)), cerebrovascular disease (0.01 (0.00064–0.16)), diabetes (360 (3.4–39,000)), thyroid disease (0.00099 (0.000012–0.085)), oligopharma (<5) (0.033 (0.0016–0.67)), and the use of zolpidem (0.000014 (0.0000000021–0.092)), antidiabetics (0.00015 (0.00000042–0.051)), diuretics (0.0003 (0.000004–0.022)), and opiates (0.000069 (0.00000035–0.013)).

## 4. Discussion

We live in a time of increasingly aged population, so we face the conflicting goals of an elderly quality of care improvement versus the optimization of health resources. In such a dilemma, there is a strong requirement for correct identification of frailty risks. However, the scientific community has not yet reached a consensual definition of frailty categories. It is not uncommon that studies take into account the frail and pre-frail categories [47,48] of Fried’s frailty phenotype. Our dataset is composed of 68.54% frail and 29.14% pre-frail patients. For this study we discard the remaining 2% robust patients.

Identification of frailty risk factors by means of the use of logistic regression to discriminate frail from pre-frail patients finds that they are associated with reduced physical capacity, such as the need walking stick, reduced mobility, weight loss, and a history of falls. Falls are an indicator of impeded daily life issues in the elderly population [49]. They have been identified by the WHO [50] as the second worldwide cause of death due to involuntary injuries, increasing their incidence with age [51]. Specifically, our results point to the performance in MNA mobility, MNA weight loss 3 months, and SPPB sit and stand up tests as frailty risk indicators, in good agreement with studies [52,53,54] that point to slowness in walking in conjunction with limited capacity to perform routine physical activities as biomarkers of the onset of frailty. These indicators, together with a smaller calf circumference, and weight loss have been confirmed as significant frailty risk factors [55]. In the cognitive aspect factors such as depression, dementia or delirium, also known as the three D’s of geriatric psychiatry [56] are factors that elderly patients can often present simultaneously [57]. In our study, however, cognitive aspects such as dementia, delirium and depression were marginally significant factors.

Systematic reviews [32,58] have found that frailty in older people [19] is a strong predictor of mortality, even independently of the frailty scale used [59]. Increases in the frailty index were associated with increased mortality, while decreases had no effect on mortality [60,61,62]. Large cohort retrospective studies [63,64,65] provide confirmation that frailty has a significant time-independent effect associated with all-cause and cause-specific mortality, with some exceptions in men. A retrospective study in Australia [66] reports interaction effects of multimorbidity and frailty that increase the mortality and adverse outcome risks for the elderly (age 75+) admitted at the hospital. These interactions effects have also been found in older people living in the community [67].

A sex analysis of frailty in humans and preclinical animal models [68] found that female sex individuals are more susceptible to frailty, while males have shorter life spans. Consequently, in our study, we found a higher mortality risk for male participants. Accordingly, for patients with acute coronary syndromes, there was a strong impact of frailty on the mortality of male patients, while there was no effect for female patients [69]. However, other prospective studies [70] did not find significant differences in mortality of frail people according to sex.

In general, increasing FFI values are associated with increased mortality after many hospital interventions such as cardiopulmonary resuscitation [71], surgical emergencies [72], lung transplantation [73], major lower extremity amputation [74], hemodialysis [75,76,77], and morbidities such as stroke [78], inflammatory bowel disease [79], and cancer [80] (although cancer postoperative mortality was found to be uncorrelated with frailty in [81]). A protective effect of frailty was found in hypertension mortality risk [82]. Similarly, depression was associated with increased mortality in pre-frail subjects, but not in frail and non-frail subjects [83]. However, in a cohort of patients with depressive disorder, frailty was found to increase mortality [84].

Malnutrition has a key role both in frailty pathogenesis [85], and as a risk of increased mortality in already frail persons [86]. An eight-year follow-up study [87] has found that nutritional frailty, assessed by a novel five-item construct, were at higher risk for all-cause mortality. In our study, we found that MNA scores have a strong association with high HR in the pre-frail cohort, while there was not an appreciable association with HR in the frail cohort.

On average, our patients have a Charlson comorbidity index of 6.39, which, according to [88], corresponds to less than 0.2 10-year survival probability. Hospital admittance was due to infectious causes, exacerbations of heart failure, and delirium. Infectious diseases account for 33% of deaths for persons aged 65 years or more [89].

We would like to highlight that the Cox regression analysis in the frail and pre-frail cohorts has produced very different sets of variables with high HRs in each cohort, having a small subset in common. This fact shows that geriatric patients have a wide variety of characteristics that need to be taken into consideration following a personalized medicine approach [90,91,92,93]. A recent study showed that An SPPB score less than 10 is predictive of mortality, thus systematic implementation of the SPPB in clinical practice settings can provide useful prognostic information on the risk of all-cause mortality [94].

Chronic heart failure (CHF) has been recognized as a geriatric syndrome [95]. HF deteriorates the patient’s quality of life substantially among the first ranked causes of morbidity and mortality in world health reports [96]. The association of CHF and frailty increases death and hospitalization hazard risks [97]. Conversely, social frailty increases both re-hospitalization and mortality due to heart failure [98]. CHF treatment commonly includes overdosing diuretics in order to recover from acute decompensation, as much as two- to three-fold the maintenance therapy doses. A review of inappropriate diuretic use has uncovered that it is a prevalent cause of death [99]. Besides, immobility, which is very common in frail patients, is a risk factor for venous thrombosis. Our findings regarding venous thrombosis as a high hazard risk in pre-frail patients agrees with studies reporting higher venous thrombosis risks for frail and pre-frail people [100]. Frailty was also found associated with increased deaths in ST-segment elevation myocardial infarction [101], and acute coronary syndrome [102].

Cerebrovascular disease (CVA) is a major cause of disability whose prevalence increases with age. In our study, we found that CVA is a significant hazardous risk in the pre-frail cohort. CVA related functional limitations are similar to those of frail elderly people. Namely, post-stroke fatigue is associated with reduced quality of life and greater risk of death [103,104].

Another disease with a high prevalence in older patients is diabetes, which causes further deterioration of physical performance in the frail population [105]. Older adults with diabetes have a high risk of recurrent hypoglycemia, a condition associated with marked morbidity and mortality [106]. We have found that diabetes is associated with high HR in the pre-frail cohort.

The physiological changes correlated with aging [107] produce the coexistence of multiple diseases treated with a large number of drugs, increasing the risk of suffering adverse drug reactions and interactions among drugs, therefore increasing mortality risk due to polypharmacy. The PPIs, indicated for gastric protection, stand out among the most prescribed drugs in western health care systems. However, prolonged use of PPIs is a cause of serious systemic side effects [108], including *Clostridium difficile* associated colitis, community acquired pneumonia, acute interstitial nephritis, vitamin B12 deficiency, and increased risk of hip fractures. Recent analyses suggest that PPIs can be inappropriately prescribed in 50% to 80% of patients, and it has been associated with an increased risk of cardiovascular disease and chronic kidney disease leading to excess mortality [109].

Elderly patients have increased risk of both ischemic and hemorrhagic events leading to increasing mortality rates [110], thus requiring careful antiplatelet management, hence our finding of antiplatelets as a hazard risk, for frail patients. Another pharmacological finding, that of quetiapine as a significant hazard risk is in agreement with literature reports of mortality of up to 2% associated with Quetiapine use [111]. Another hypnotic drug, zolpidem, is associated with a higher risk of mortality according to a recent study [112].

One clear result of our study is that polypharmacy [113] poses a strong risk of death at two years after recruitment. Medication management in older people experiencing multimorbidity and the risk of prescription cascades are often challenging. The analysis of the adequacy of treatment or the identification of potentially inappropriate drugs (MPI) has not been defined as the objective of this study. It has been considered that it is the responsibility of the doctor who has ultimately indicated the drugs to be aware and to adapt the treatment to the situation that the patients have at that moment. Possibly, the most interesting option for the patient is to adapt the treatment to their state of fragility while closely monitoring the results. However, as multimorbidity patients are treated by a diversity of specialists, it will be necessary to create multidisciplinary teams to manage the cases where serious adverse events may emerge from the interaction between several drugs.

### Limitations

One limitation is that the cohort was recruited from only one site. Expansion of the study in several places would be recommended to confirm some of the conclusions, but this is difficult to achieve due to a lack of consensus in the clinical community about the information collected at the time of admission.

The study is an observational investigation that was not designed to assess a cause and effect relationship between frailty and clinical outcomes, although the multivariate analysis was designed to account for these as covariates.

At hospital discharge, it was not feasible to repeat the physical and cognitive evaluations due to the discharge procedures; thus, it was not possible to evaluate the recovery of the conditions that classify a patient with frailty.

We used the Fried phenotype to characterize frailty status, while there are currently diverse efforts to define more accurate frailty indices based on multimorbidity identified by ICD-10 codes [114] and other biomarkers [115,116] that should be used in future studies. We did not carry out a sex analysis, which can be the subject of future work.

In this study, we evaluated the frailty state at recruitment time, but we were unable to carry out follow up of the evolution of the subjects’ frailty conditions to assess recovery to a robust condition. Such dynamic frailty analysis will be desirable in future studies.

## 5. Conclusions and Future Work

In this study, we assessed mortality risk after two years of follow up of frail versus pre-frail patients admitted to the hospital. Frailty was assessed according to the Fried phenotype. We found that frailty is associated with increased mortality at two years by comparison of the mortality curves and long-rank test, in agreement with the increasing body of evidence on the risks of frailty.

This study also confirmed that some comorbidities such as congestive heart failure, deep venous thrombosis, cerebrovascular disease, and diabetes appear as high risk factors for mortality in frail and pre-frail patients. In addition, the use of some drugs such as PPIs, antiplatelet, quetiapine, paracetamol, zolpidem, antidiabetics, diuretics, and opiates is associated with a higher risk of mortality. In this study, the frail and pre-frail categories are clearly separated with specific risk variables for each one, which points to the need of personalized approaches in their treatment.

## Figures and Tables

**Table 1:** Significant variables (p<0.05) identified in multivariate logistic regression discriminating frail from pre-frail subjects.

	Estimate	Standard Error	Standardized	Odds Ratio	z	Wald Statistic	df	*p*	95% CI
Needs Walking Stick	0.842	0.318	0.392	2.321	2.649	7.019	1	0.008	(0.219, 1.465)
MNA Weight Loss 3 months	0.683	0.197	0.812	1.980	3.467	12.019	1	<0.001	(0.297, 1.070)
MNA Mobility	2.165	0.599	1.029	8.717	3.612	13.046	1	<0.001	(0.990, 3.340)
SPPB March 4 m	0.590	0.210	0.590	1.804	2.810	7.897	1	0.005	(0.179, 1.002)
Falls	−0.618	0.319	−0.296	0.539	−1.936	3.749	1	0.053	(−1.243, 0.008)
Dementia	2.146	0.965	0.436	8.552	2.225	4.950	1	0.026	(0.256, 4.037
Depression	1.641	0.780	0.465	5.162	2.104	4.427	1	0.035	(0.112, 3.171)
Delirium	−0.734	0.370	−0.325	0.480	−1.987	3.947	1	0.047	(−1.459, −0.010)

Note: CI—confidence interval.

**Table 2:** Variables with significant (p<0.05) high risk (HR, 95% Confidence Interval) found by Cox’s regression carried out over the significant variables identified by univariate *t*-tests. Frail cohort (N = 501), data censored at 2 years.

	2 Years					
Variable	Coefficients	Exp(Coef)	Se(Coef)	z	HR	*p*
Age	0.048	1.049	0.019	2.545	1.05 (1.01–1.09)	0.011
Sex	−0.817	0.442	0.207	−3.945	0.44 (0.29–0.66)	<0.001
Weight	−0.019	0.980	0.009	−2.218	0.98 (0.96–1.00)	0.027
R30	−0.703	0.495	0.203	−3.463	0.50 (0.33–0.74)	<0.001
SPPB-SUG	−0.482	0.618	0.162	−2.980	0.62 (0.45–0.85)	0.003
Congestive Heart Failure	−0.398	0.671	0.193	−2.062	0.67 (0.46–0.98)	0.039
PPIs	−0.512	0.599	0.188	−2.722	0.60 (0.41–0.87)	0.006
Antiplatelet	0.588	1.800	0.209	2.808	1.80 (1.19–2.71)	0.005
Quetiapine	−1.166	0.312	0.488	−2.387	0.31 (0.12–0.81)	0.017
Paracetamol	0.368	1.445	0.187	1.973	1.45 (1.00–2.08)	0.049

Note: Coef—coefficient; Exp(Coef)—exponential coefficients; Se(Coef)—standard error; z—z score; R30— patient experienced readmission in the 30 days after discharge; SPPB-SUG—sit and stand up from the SPPB test; PPIs—proton-pump inhibitors.

**Table 3:** Variables with significant (p<0.05) high risk (HR, 95% Confidence Interval) found by Cox’s regression carried out over the significant variables identified by by univariate *t*-tests. Pre-frail cohort: (N = 213), data censored at 2 years.

	2 Years					
Variable	Coefficients	Exp(Coef)	Se(Coef)	z	HR	*p*
Age	0.518	1.679	0.131	3.945	1.7 (1.3–2.2)	<0.001
Sex	−5.295	0.005	1.945	−2.723	0.005 ( 0.00011–0.23)	0.006
Weight	0.251	1.286	0.066	3.806	1.3 (1.1–1.5)	<0.001
Own-home	4.824	124.4	2.065	2.336	120 (2.2–7100)	0.019
Barthel	4.748	115.3	1.383	3.434	120 (7.7–1700)	<0.001
Pfeiffer	2.131	8.426	0.669	3.184	8.4 (2.3–31)	0.001
MNA	7.127	1246	2.174	3.278	1200 (18–88,000)	0.001
MNA Weight Loss 3 months	2.410	11.14	0.815	2.957	11 (2.3–55)	0.003
MNA Mobility	6.378	588.7	2.043	3.123	590 (11–32,000)	0.002
MNA Acute Disease 3 months	−7.656	<0.001	2.038	−3.757	0.00047 (0.0000087–0.026)	<0.001
Vision Loss	−3.160	0.042	1.247	−2.534	0.042 (0.0037–0.49)	0.011
Constipation	−3.687	0.025	1.143	−3.226	0.025 (0.0027–0.24)	0.001
Falls	9.797	1798	2.444	4.009	18,000 (150–2,200,000)	<0.001
Congestive Heart Failure	3.739	42.07	1.761	2.124	42 (1.3–1300)	0.034
Deep Venous Thrombosis	9.038	8421	3.098	2.917	8400 (19–3,700,000)	0.004
Cerebrovascular Disease	−4.585	0.010	1.409	−3.255	0.01 (0.00064–0.16)	0.001
Diabetes	5.899	364.8	2.385	2.474	360 (3.4–39,000)	0.013
Thyroid Disease	−6.913	<0.001	2.271	−3.045	0.00099 (0.000012–0.085)	0.002
Drug Oligopharma	−3.410	0.033	1.537	−2.219	0.033 (0.0016–0.67)	0.026
PPIs	−2.780	0.062	1.101	−2.524	0.062 (0.0072–0.54)	0.012
Zolpidem	−11.19	<0.001	4.488	−2.493	0.000014 (0.0000000021–0.092)	0.013
Antidiabetics	−8.830	<0.001	<0.001	−2.953	0.00015 (0.00000042–0.051)	0.003
Diuretics	−8.113	<0.001	2.196	−3.694	0.0003 (0.000004–0.022)	<0.001
Opiates	−9.585	<0.001	2.691	−3.562	0.000069 (0.00000035–0.013)	<0.001

Note: Coef—coefficient; Exp(Coef)—exponential coefficients; Se(Coef)—standard error; z—standard score; PPIs—proton-pump inhibitors, Drug Oligopharma—take less than 5.

## Data Availability

The anonymized data have been published in zenodo.org at https://zenodo.org/record/5803234.

## Supplementary Materials

The following supporting information can be downloaded at: https://www.mdpi.com/article/10.3390/jcm12093103/s1, Figure S1: Hazard ratios for the frail cohort, data censored at 2 years; Figure S2: Hazard ratios for the pre-frail cohort, data censored at 2 years; Table S1: Independent Samples T-Test; Table S2: Logistic Regression.
