# Supplementary material for: Mortality Risks after Two Years in Frail and Pre-Frail Older Adults Admitted to Hospital
Source: J Clin Med. 2023 Apr 24;12(9):3103. doi: 10.3390/jcm12093103 (PMC10179017; doi:10.3390/jcm12093103)
Supplement: Supplementary file 1 [file jcm-12-03103-s001.zip › jcm-2180392-supplementary.pdf]

Supplementary material for "MORTALITY RISKS IN FRAIL and PRE-FRAIL OLDER ADULTS ADMITTED TO HOSPITAL AFTER TWO YEARS"

January 3, 2023

Table S1: Independent Samples T-Test

Independent Samples T-Test

	t	df	p
Age	6.365	712	< .001 [a]
Gender	3.616	712	< .001 [a]
Weight	-3.478	696	< .001
Readmission30	-3.429	712	< .001 [a]
Married	0.448	712	0.654
Single	0.635	712	0.525
Divorced	-0.537	712	0.592
Widowed	-3.619	712	< .001 [a]
Descendency	-0.547	556	0.585
Needswalkingstick	-8.387	592	< .001 [a]
Ownhome	3.382	617	< .001 [a]
Alone	1.600	622	0.110 [a]
Othershome	-1.695	612	0.091 [a]
Retirementhouse	-2.613	613	0.009 [a]
Barthel	-8.359	656	< .001
Pfeiffer	3.938	709	< .001 [a]
MNA	9.864	712	< .001
MNAWeightloss3m	-7.555	712	< .001 [a]
MNAMobility	-8.444	712	< .001 [a]
MNAAcutedisease3m	-2.861	711	0.004 [a]
MNANeuropsychologicalproblems	-2.006	712	0.045 [a]
MNACalfcircumference	-4.068	712	< .001 [a]
MNAAppetiteloss3m	-5.801	712	< .001 [a]
SPPB	-12.708	711	< .001 [a]
SPPBSitandgetup	-10.154	710	< .001 [a]
SPPBBalance	-8.689	709	< .001
SPPBMarch4m	-14.559	707	< .001 [a]
Hearingloss	-3.194	712	0.001 [a]
Visionloss	-2.088	712	0.037 [a]
Constipation	-3.710	712	< .001 [a]
Falls	-3.453	712	< .001 [a]
Dysphagia	-3.444	712	< .001 [a]
AHT	-1.540	712	0.124 [a]
Cho	0.567	712	0.571
Smoker	0.425	712	0.671
Exsmoker	1.350	712	0.178 [a]
Arrhythmia	-2.044	712	0.041 [a]
Syncope	0.812	712	0.417
Headtrauma	-1.045	712	0.297 [a]
AMI	1.026	712	0.305 [a]
Congestiveheartfailure	-3.573	712	< .001 [a]

Independent Samples T-Test

	t	df	p
Peripheralvasculardisease	1.418	712	0.157 [a]
Deepvenousthrombosis	1.969	712	0.049 [a]
Pulmonaryembolism	1.007	712	0.314 [a]
Cerebrovasculardisease	-2.407	712	0.016 [a]
Dementia	-1.986	712	0.047 [a]
COPDAsthma	-0.259	712	0.796
Connectivetissuepathology	-1.866	712	0.062 [a]
Ulcerdisease	0.558	712	0.577
Diabetes	-2.738	712	0.006 [a]
Diabeteswithorgandamage	-1.399	712	0.162 [a]
Kidneypathology	-2.207	712	0.028 [a]
Ooncology	1.279	712	0.201 [a]
Depression	-2.563	712	0.011 [a]
Mooddisorder	-2.545	712	0.011 [a]
Osteo	-1.753	712	0.080 [a]
Thyroid	-2.244	712	0.025 [a]
Epilepsy	0.074	712	0.941
Glaucoma	-1.399	712	0.162 [a]
Delirium	-2.074	712	0.038 [a]
DrugOligopharma	4.004	712	< .001 [a]
DrugModerate	-1.066	712	0.287 [a]
DrugSevere	-2.547	712	0.011 [a]
IBP	-2.360	712	0.019
Antiplatelet	1.984	712	0.048 [a]
Hypnotic	-3.318	712	< .001 [a]
Benzodiazapine	-2.701	712	0.007 [a]
Zolpidem	-2.164	712	0.031 [a]
Neuroleptics	-2.297	712	0.022 [a]
Quetiapine	-2.189	712	0.029 [a]
Risperdal	-0.482	712	0.630
Otherantipsychotics	-0.270	712	0.787
Antiepileptic	-0.739	712	0.460
Antiparkinsonian	-0.850	712	0.395
AISRS	-2.323	712	0.020 [a]
ATricyclic	-0.187	712	0.852
AHeterocyclic	-2.442	712	0.015 [a]
ADual	1.084	712	0.279 [a]
Insulin	-1.533	712	0.126 [a]
Arrhythmics	-3.095	712	0.002 [a]
Digoxin	-1.657	712	0.098 [a]
Antiarrhythmic	-0.149	712	0.882
Calciumantagonist	-0.861	712	0.389
ACEinhibitor	0.984	712	0.326

Independent Samples T-Test

	t	df	p
BAT1	0.810	712	0.418
Betablocker	-0.993	712	0.321 [a]
NitratesNitroglycerin	1.009	712	0.313 [a]
Diuretics	-3.415	712	< .001
Lipidmodifier	1.323	712	0.186 [a]
Inhaled	-1.369	712	0.171 [a]
Genitourinary	-0.740	712	0.460
Antagoalfaadrenerg	0.530	712	0.596
Inhtest5alfa	-0.876	712	0.381
Antispasmodics	-0.569	712	0.569
Uricosurics	-0.666	712	0.506
Laxatives	-1.745	712	0.081 [a]
Opiates	-2.986	712	0.003 [a]
Paracetamol	-2.116	712	0.035 [a]
OtherNSAIDs	-1.262	712	0.207 [a]
Ophthalmus	-0.918	712	0.359
Anticoagulated	-2.018	712	0.044 [a]
Antidepressant	-3.647	712	< .001 [a]
Antidiabetics	-2.291	712	0.022 [a]
Alphaadrenergic	0.686	712	0.493

Note. Student's t-test.

[a] Levene's test is significant ($p < .05$), suggesting a violation of the equal variance assumption

Table S2: Logistic Regression

Model Summary - Fried

Model	Deviance	AIC	BIC	df	X^2	p	McFadden R^2	Nagelkerke R^2	Tjur R^2	Cox & Snell R^2
H_0	608.143	610.143	614.423	533						
H_1	388.856	500.856	740.559	478	219.286	< .001	0.361	0.495	0.396	0.337

Coefficients

						Wald Test			95% Confidence interval	
	Estimate	Standard Error	Standardized⁺	Odds Ratio	z	Wald Statistic	df	p	Lower bound	Upper bound
(Intercept)	-12.338	4.690	-1.987	4.380e-6	-2.631	6.922	1	0.009	-21.530	-3.147
Age	-0.020	0.028	-0.123	0.980	-0.715	0.511	1	0.475	-0.076	0.035
Gender	-0.357	0.350	-0.179	0.700	-1.021	1.042	1	0.307	-1.043	0.329
Weight	0.010	0.014	0.134	1.010	0.704	0.496	1	0.481	-0.018	0.038
Readmission30	0.628	0.389	0.236	1.874	1.615	2.608	1	0.106	-0.134	1.391
Widowed	0.010	0.308	0.005	1.010	0.033	0.001	1	0.973	-0.594	0.615
Needswalkingstick	0.842	0.318	0.392	2.321	2.649	7.019	1	0.008	0.219	1.465
Ownhome	-0.137	0.517	-0.052	0.872	-0.264	0.070	1	0.792	-1.150	0.877
Retirementhouse	0.054	0.698	0.016	1.055	0.077	0.006	1	0.939	-1.314	1.421
Barthel	0.256	0.283	0.180	1.291	0.904	0.817	1	0.366	-0.299	0.810

Model Summary - Fried

Model	Deviance	AIC	BIC	df	X²	p	McFadden R²	Nagelkerke R²	Tjur R²	Cox & Snell R²
Pfeiffer	0.170	0.212	0.159	1.185	0.800	0.641	1	0.423	-0.246	0.585
MNA	0.501	0.470	0.348	1.651	1.067	1.139	1	0.286	-0.419	1.421
MNAWeightloss3m	0.683	0.197	0.812	1.980	3.467	12.019	1	< .001	0.297	1.070
MNAMobility	2.165	0.599	1.029	8.717	3.612	13.046	1	< .001	0.990	3.340
MNAAcutedisease3m	0.395	0.372	0.166	1.484	1.061	1.127	1	0.289	-0.334	1.124
MNANeuropsychologicalproblems	-0.597	0.447	-0.254	0.551	-1.336	1.785	1	0.182	-1.472	0.279
MNACalfcircumference	0.181	0.181	0.236	1.198	0.996	0.993	1	0.319	-0.175	0.536
MNAAppetiteloss3m	0.291	0.247	0.220	1.338	1.177	1.386	1	0.239	-0.194	0.776
SPPB	0.092	0.456	0.087	1.096	0.201	0.040	1	0.841	-0.802	0.986
SPPBSitandgetup	0.175	0.226	0.196	1.192	0.776	0.602	1	0.438	-0.268	0.619
SPPBBalance	-0.013	0.190	-0.017	0.987	-0.067	0.004	1	0.947	-0.385	0.360
SPPBMarch4m	0.590	0.210	0.590	1.804	2.810	7.897	1	0.005	0.179	1.002
Hearingloss	-0.331	0.344	-0.165	0.718	-0.961	0.924	1	0.336	-1.006	0.344
Visionloss	-0.150	0.354	-0.073	0.860	-0.425	0.181	1	0.671	-0.844	0.543
Constipation	0.350	0.311	0.167	1.420	1.126	1.268	1	0.260	-0.259	0.960
Falls	-0.618	0.319	-0.296	0.539	-1.936	3.749	1	0.053	-1.243	0.008
Dysphagia	0.487	0.535	0.163	1.627	0.909	0.827	1	0.363	-0.562	1.535
Arrhythmia	-0.203	0.436	-0.100	0.816	-0.467	0.218	1	0.641	-1.057	0.651
Congestiveheartfailure	0.490	0.349	0.229	1.632	1.404	1.972	1	0.160	-0.194	1.174

Model Summary - Fried

Model	Deviance	AIC	BIC	df	X^2	p	McFadden R^2	Nagelkerke R^2	Tjur R^2	Cox & Snell R^2
Deepvenousthrombosis	-0.928	0.607	-0.189	0.395	-1.529	2.339	1	0.126	-2.117	0.261
Cerebrovasculardisease	0.161	0.412	0.059	1.174	0.390	0.152	1	0.696	-0.647	0.968
Dementia	2.146	0.965	0.436	8.552	2.225	4.950	1	0.026	0.256	4.037
Diabetes	0.790	0.551	0.374	2.204	1.434	2.057	1	0.152	-0.290	1.870
Kidneypathology	-0.219	0.358	-0.089	0.804	-0.610	0.372	1	0.542	-0.921	0.484
Depression	1.641	0.780	0.465	5.162	2.104	4.427	1	0.035	0.112	3.171
Mooddisorder	1.336	0.745	0.364	3.804	1.793	3.214	1	0.073	-0.125	2.797
Thyroid	0.211	0.456	0.072	1.235	0.463	0.214	1	0.644	-0.682	1.104
Delirium	-0.734	0.370	-0.325	0.480	-1.987	3.947	1	0.047	-1.459	-0.010
DrugOligopharma	-0.584	0.386	-0.236	0.557	-1.514	2.291	1	0.130	-1.341	0.172
DrugSevere	0.083	0.404	0.039	1.086	0.204	0.042	1	0.838	-0.710	0.875
IBP	-0.270	0.303	-0.135	0.763	-0.890	0.793	1	0.373	-0.865	0.324
Antiplatelet	-0.329	0.343	-0.150	0.719	-0.959	0.920	1	0.338	-1.002	0.344
Hypnotic	2.150	1.820	1.023	8.586	1.181	1.396	1	0.237	-1.417	5.717
Benzodiazapine	-2.012	1.799	-0.937	0.134	-1.119	1.251	1	0.263	-5.537	1.513
Zolpidem	-0.589	1.353	-0.114	0.555	-0.435	0.189	1	0.664	-3.240	2.063
Neuroleptics	0.127	0.642	0.036	1.136	0.198	0.039	1	0.843	-1.131	1.386

Model Summary - Fried

Model	Deviance	AIC	BIC	df	X²	p	McFadden R²	Nagelkerke R²	Tjur R²	Cox & Snell R²
Quetiapine	1.064	1.511	0.176	2.898	0.704	0.496	1	0.481	-1.898	4.026
AISRS	-1.367	1.208	-0.391	0.255	-1.131	1.279	1	0.258	-3.735	1.002
AHeterocyclic	-0.651	1.176	-0.204	0.521	-0.554	0.306	1	0.580	-2.957	1.654
Arrhythmics	0.672	0.404	0.268	1.958	1.664	2.770	1	0.096	-0.119	1.463
Diuretics	-0.140	0.311	-0.069	0.869	-0.451	0.203	1	0.652	-0.750	0.469
Opiates	0.563	0.444	0.192	1.756	1.267	1.606	1	0.205	-0.308	1.434
Paracetamol	-0.103	0.304	-0.050	0.902	-0.339	0.115	1	0.734	-0.699	0.492
Anticoagulated	-0.147	0.444	-0.072	0.863	-0.331	0.109	1	0.741	-1.017	0.723
Antidepressant	0.509	1.089	0.214	1.664	0.468	0.219	1	0.640	-1.625	2.644
Antidiabetics	-0.615	0.591	-0.274	0.541	-1.041	1.084	1	0.298	-1.773	0.543

Note. Fried level '1' coded as class 1.

$^+$ Standardized estimates represent estimates where the continuous predictors are standardized (X-standardization).

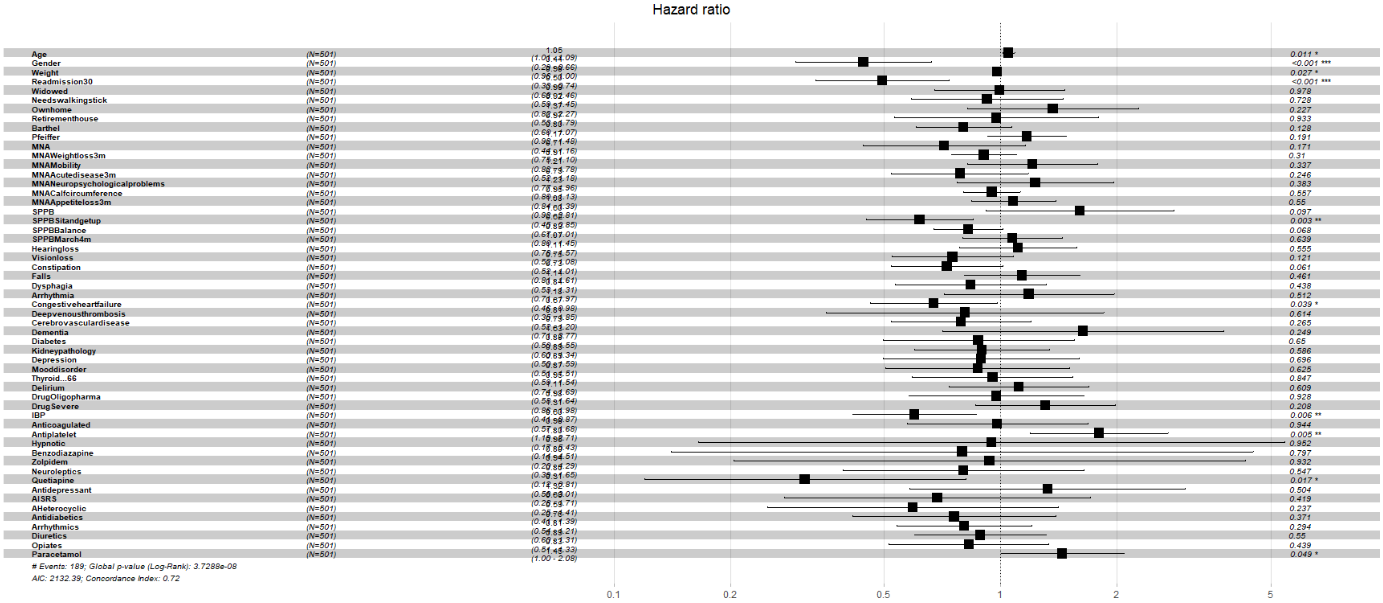

Figure S1: Hazard ratios for the frail cohort, data censored at 2 years.

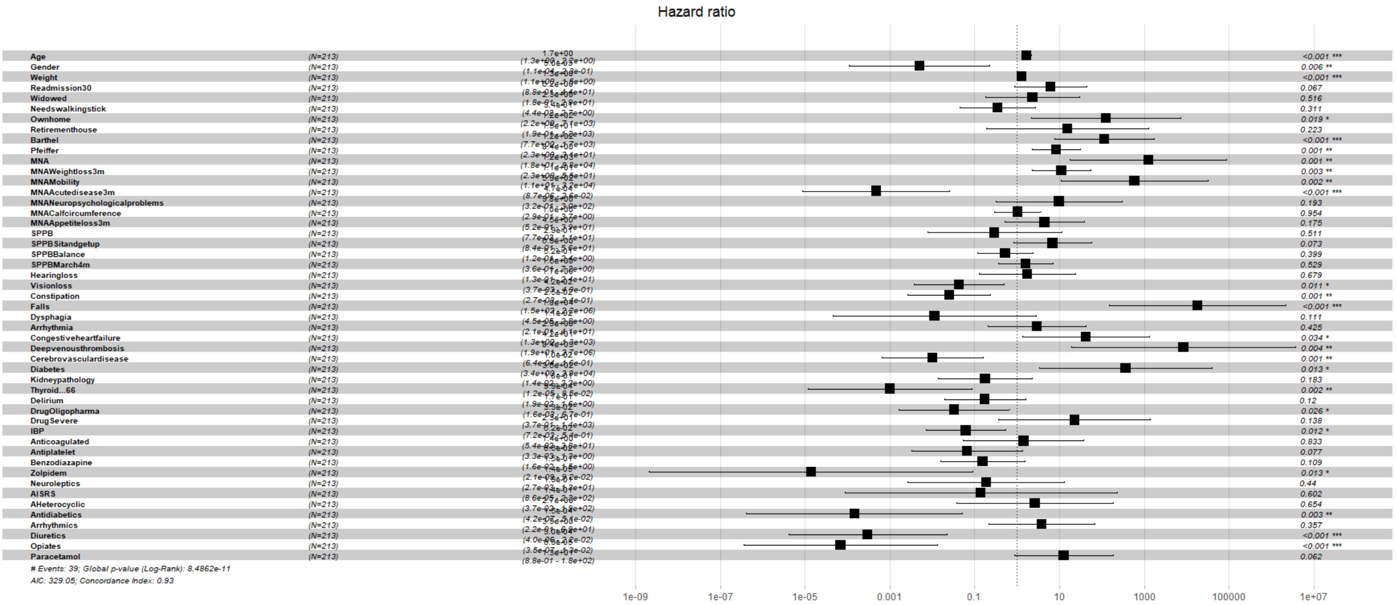

Figure S2: Hazard ratios for the pre-frail cohort, data censored at 2 years.
